# Effect of probiotic supplementation in nonalcoholic steatohepatitis patients: PROBILIVER TRIAL protocol

**DOI:** 10.1186/s13063-019-3679-7

**Published:** 2019-10-10

**Authors:** Amanda Souza Silva-Sperb, Helena Abadie Moraes, Bruna Concheski de Moura, Bruna Cherubini Alves, Juliana Paula Bruch-Bertani, Vittoria Zambon Azevedo, Valesca Dall’Alba

**Affiliations:** 1 Graduate Program: Sciences of Gastroenterology and Hepatology, Universidade Federal do Rio Grande do Sul (UFRGS), Rua Ramiro Barcelos 2400 - 2nd floor, Porto Alegre, RS 90035-003 Brazil; 2 Department of Nutrition, Universidade do Vale do Taquari, Lajeado, Brazil; 3 Graduate Program in Food, Nutrition and Health, UFRGS, Porto Alegre, Brazil; 4 Nutrition and Dietetics Division, Hospital de Clínicas de Porto Alegre, Porto Alegre, Brazil; 5 Department of Nutrition, School of Medicine, UFRGS, Porto Alegre, Brazil

**Keywords:** Non-alcoholic fatty-liver disease, Probiotic, Sarcopenia, Inflammation, Microbiota, Permeability

## Abstract

**Background:**

Recently factors in the relationship between gut microbiota, obesity, diabetes and the metabolic syndrome have been suggested in the development and progression of nonalcoholic steatohepatitis (NASH). In this sense, this work aims to evaluate the effects of probiotic supplementation on intestinal microbiota modulation, degree of hepatic steatosis and fibrosis, inflammation, gut permeability, and body composition.

**Methods:**

This double-blind, randomized clinical trial will include adult outpatients with a diagnosis of NASH confirmed by biopsy with or without transient elastography. All patients will undergo a complete anamnesis to investigate their alcohol consumption, previous history, medications, nutritional assessment (dietary intake and body composition), sarcopenia, physical activity level and physical and functional capacity, cardiovascular risk, biochemical parameters for assessment of inflammatory status, lipid profile, hepatic function, gut permeability, and assessment of microbiota. These procedures will be performed at baseline and repeated after 24 weeks (at the end of the study). Through the process of randomization, patients will be allocated to receive treatment A or treatment B. Both patients and researchers involved will be blinded (double-blind study). The intervention consists of treatment with a probiotic mix (*Lactobacillus acidophillus* + *Bifidobacterium lactis* + *Lactobacillus rhamnosus* + *Lactobacillus paracasei*, 1 x 10^9^ CFU for each) and the placebo which is identical in all its characteristics and packaging. Patients will be instructed to consume two sachets/day during 24 weeks and to report any symptoms or side effects related to the use of the sachets. Adherence control will be carried out through the patient’s notes on a form provided, and also by checking the number of sachets used.

**Discussion:**

The final results of study will be analyzed and disseminated in 2020.

**Trial registration:**

ClinicalTrials.gov, ID: NCT03467282. Registered on 15 March 2018.

**Electronic supplementary material:**

The online version of this article (10.1186/s13063-019-3679-7) contains supplementary material, which is available to authorized users.

## Background

According to the American Association for the Study of Liver Diseases, non-alcoholic fatty-liver disease (NAFLD) is defined as the presence of steatosis, confirmed by imaging or histology, and not caused by significant alcohol consumption, long-term use of a steatogenic medication, or a monogenic hereditary disorder. Histologically, NAFLD can be categorized into nonalcoholic fatty liver (NAFL) or non-alcoholic steatohepatitis (NASH), defined as the presence of ≥ 5% of steatosis and inflammation with hepatocyte injury, with or without fibrosis [1]. By being highly associated with metabolic comorbidities, such as obesity, diabetes mellitus, and dyslipidemia, NAFLD is considered a hepatic manifestation of the metabolic syndrome [2, 3].

The global prevalence of NAFLD diagnosed by imaging is estimated at around 25% [4]. This liver disease is positively related to the epidemic of obesity, and metabolic diseases. When NASH is diagnosed via liver biopsy, a procedure not always feasible in the studies, the estimated prevalence in the general population varies from between 1.5 and 6.4%. Patients with NASH with a higher degree of fibrosis and concurrent metabolic diseases, such as diabetes, hypertension, visceral obesity, and dyslipidemia, present a higher mortality risk. Therefore, patients with this profile have a greater requirement for treatment [1].

The gut microbiota seems to be related to NAFLD/NASH by contributing to intestinal permeability and choline-metabolism disturbance, endogenous-alcohol production, inflammatory cytokine release, and hepatic Toll-like receptor (TLR) regulation. Moreover, some studies have suggested that patients with NASH have a different microbiota composition than patients without the disease [5].

Microbiota dysbiosis is related to dysfunction of the intestinal barrier, increasing its permeability and exposing the liver to microbial translocation [6, 7]. The increased permeability allows the passage of products from the gut microbiota into the portal circulation, stimulating an inflammatory cascade and favoring the development of NASH, hepatic fibrosis, and hepatocellular carcinoma [8–10]. The relation of the intestine-liver axis in an environment of increased intestinal permeability seems to play an important role in the pathogenic mechanism of NASH. Several studies have documented the progression of NASH related to increased intestinal permeability [6, 8, 11–14].

Also, it has been described that the gut microbiota is related to liver steatosis and inflammation involving TLR, especially TLR-4, and proinflammatory cytokines [15]. Experimental studies lead us to believe that probiotics could be considered a therapeutic option in NAFLD. In our group, the effect of the probiotic *Lactobacillus rhamnosus* GG (LGG) on a model of alcohol steatosis in zebrafish was evaluated. A reduction of serum triglycerides, cholesterol, and hepatic steatosis levels was observed in treated animals [16]. There are already clinical trials that demonstrate the benefit of probiotic supplementation by reducing liver enzyme levels, steatosis severity (measured by the Fatty Liver Index), and proinflammatory cytokines, such as tumor necrosis factor alpha (TNFɑ) and interleukin (IL)-6, and also improve the lipid profile [17–21]. However, these studies are very heterogeneous in terms of the intervention time and the strains of bacteria involved.

Another concern in NAFLD is the relationship with sarcopenia [22–24], characterized by the loss of skeletal muscle mass, muscle strength and physical performance, with adverse risks [25]. Lee et al. [24] highlight that individuals with sarcopenia have a higher risk of NAFLD and liver fibrosis development. Wijarnpreecha et al. [26] and Bindels et al. [27] suggest a relationship between muscle loss and changes in gut microbiota. The authors suggest that the modulated microbiota may act on muscle physiology through amino-acid changes, influencing metabolites, such as bile acids, and modulate the production of proinflammatory cytokines, an effect correlated with an improvement in the markers of skeletal-muscle atrophy.

Currently, there is no broad-spectrum drug to treat NASH, so, to prevent or delay the progression of liver disease, the associated comorbidities must be treated separately. However, we believe that modulating the microbiota through probiotic supplementation could confer benefit on NASH, especially by reducing inflammation, and thus becoming an adjunct therapeutic option. In this context, we have designed a randomized clinical trial in order to evaluate the effect of probiotic supplementation on microbiota and in the course of NASH.

## Methods

### Study setting

This is a single-center, randomized, double-blind, placebo-controlled clinical trial that will include adult subjects with NASH treated at an outpatient clinic of the Nutrition and Dietetic Division of Hospital de Clínicas de Porto Alegre, Brazil. The present protocol was written in accordance with Standard Protocol Items: Recommendations for Interventional Trials (SPIRIT) guidelines and completing the SPIRIT Checklist [28] (Additional file 1).

### Eligibility criteria

Inclusion criteria: adult subjects diagnosed with NASH, with or without fibrosis, by liver biopsy.

Exclusion criteria: patients with human immunodeficiency virus, hepatitis B virus or hepatitis C virus infection; a significant intake of alcohol; cirrhosis; pregnancy; liver transplantation; supplements and foods with probiotics; use of immunosuppressant, antibiotic, and corticosteroid drugs, and valproic acid and amiodarone, and with any other chronic inflammatory diseases.

### Interventions

The patients will be randomized to an intervention group (probiotic) or a control group (placebo). Patients allocated to the intervention group will receive probiotic supplementation which consists of a 1-g sachet containing *Lactobacillus acidophilus* SD5221 (1 x 10^9^ CFU) + *Lactobacillus rhamnosus* SD5675 (1 x 10^9^ CFU) + *Lactobacillus paracasei* SD5275 (1 x 10^9^ CFU) + *Bifidobacterium lactis* SD5674 (1 x 10^9^ CFU), while those allocated to the control group will receive a 1-g sachet with an identical appearance (physical and organoleptic) containing polydextrose/maltodextrin as the placebo. Patients will be instructed to ingest two sachets daily with water at room temperature for a period of 24 weeks.

The patients will receive a spreadsheet to mark the intake of the sachets and write down any symptoms that may appear during the intervention period. Every 45 days, patients will be seen and adherence to treatment will be evaluated through the spreadsheet and by counting the sachets that were not consumed.

The participants will be instructed to advise the research team about the need to use any other non-routine medications, as some medications may alter the intestinal microbiota, and also to inform the team when they use a product that contains probiotics.

### Randomization

The randomization will be performed through a simple, sequential, randomization plan generated online (using the randomization.com website).

### Outcomes

Primary outcome:

- Modification in fibrosis level by elastography scores

Secondary outcomes:

- Gut microbiota diversity assessed by metagenomic analysis
- Intestinal permeability assessed by claudin-3 and lipopolysaccharide (LPS) levels
- Inflammatory response evaluated by *TLR-4* gene expression and biomarker cytokeratin 18 (CK-18) and C-reactive protein (CRP) levels
- Components of the metabolic syndrome evaluated by anthropometry and laboratorial assessment
- Modifications in the parameters of sarcopenia evaluated by Dual-energy X-ray Absorptiometry (DEXA), bioelectrical impedance analysis (BIA), physical capacity, and myostatin, a negative regulator of muscle growth, and testosterone levels

### Participant timeline

**Fig. 1:**
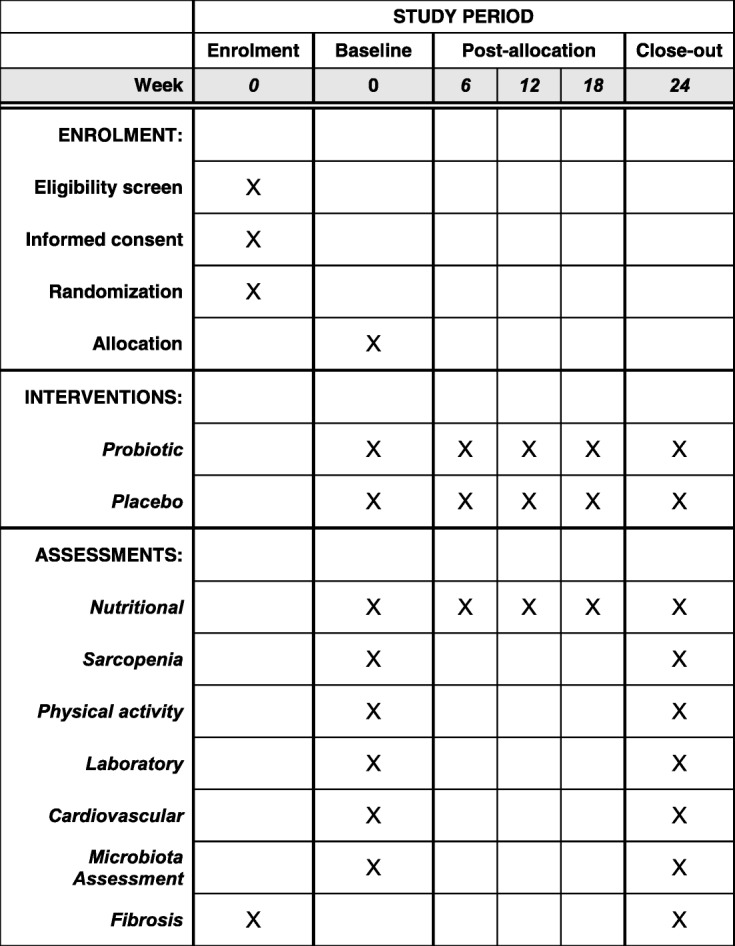
Schedule of enrollment, interventions, and assessments

The participant timeline is presented in Fig. 1.

### Sample size

The sample size estimation was carried out in WINPEPI 11.20 (Brixton Health, Israel), based on data from Eslamparast et al. [29] that found a mean reduction in fibrosis score of 9.36 ± 1.9 to 6.38 ± 1.5 in NAFLD patients taking symbiotic supplementation (*P* < 0.001, compared to placebo). Thus, considering a power of 90% and a significance of 5%, adding 10% to compensate for eventual losses, it will be necessary to include 46 patients with NAFLD in the present study.

### Recruitment

Potentially eligible patients will be identified in the everyday clinical practice of the research staff, or referred to them for assessment of eligibility having been identified by clinical staff who are not research staff. Medical records will be checked to identify any other potentially eligible patients. The patient’s eligibility will be confirmed by the responsible researcher. After confirmation, any patient who agrees to participate in the research must sign the inform consent to begin participation.

### Allocation

Patients will be allocated to group A or group B, in a numerical sequence from 1 to 46 generated using the randomization.com website. The list will be in the possession of the researchers responsible for recruitment, but only the external investigator will have access to which patients are receiving probiotic or placebo. Delivery of the supplementation will be made to the participants according to the list generated by researchers and will be communicated to the external researcher in the sequence on the list. The products are stored in boxes identified according to the researchers’ randomization list. The allocation sequence was generated, and will be administered, by the external researcher and the participants will be included and referred to the procedures by the other researchers.

### Blinding

Patients and the researchers administering the study will not know the composition of each sachet of supplements and the participant’s allocation treatment. An external researcher will be unblinded. Researchers will know which supplements each participant received only at the end of the study. The external researcher will be informed about the composition of each supplement if needed.

### Data collection methods

The evaluations will be carried out by three trained researchers: two registered nutritionist dietitians and one undergraduate student.

#### Clinical evaluation

The data collection protocol includes demographic data, clinical diagnosis and diagnostic methods, medications in use, alcohol consumption, smoking status, and blood pressure measurement. In addition, details of the disease diagnosis (liver biopsy and transient elastography) are recorded.

#### Diagnosis

The patients were, or will be, diagnosed with NASH by liver biopsy, which is the gold standard procedure. Biopsies were, or will be, performed at Hospital de Clínicas de Porto Alegre (HCPA) or another institution, requested in the outpatient clinic as needed and medically indicated. The procedure is invasive and requires some care, but the recovery is fast. A fragment of liver is removed for analysis and used for classification as not NAFLD (< 5% of hepatic steatosis), NAFL (≥ 5% of hepatic steatosis without evidence of hepatocellular injury in the form of hepatocyte ballooning), and NASH (≥ 5% of hepatic steatosis and inflammation with hepatocyte injury, with or without any fibrosis). Liver biopsy results are also scored by the NAFLD Activity Score (NAS), which is a tool for measuring change in liver histology [1].

#### Hepatic structure and funcional assessment

Liver fibrosis will be assessed by transient elastography (FibroScan). The Controlled Attenuation Parameter (CAP) is a tool based on the FibroScan, used for the detection and quantification of liver steatosis. The elastic wave propagates through the liver at a certain rate dependent on hepatic stiffness (fibrosis). The higher the velocity, the greater the stiffness, measured in kilopascals (kPa), and the greater the extent of the fibrosis. The median is considered the most representative value, being the immediate result, and is numerical and expressed in kPa [30].

In addition, blood samples will be collected for the evaluation of the genetic expression of biomarker *TRL4*, and CK-18, which are directly associated to liver inflammation.

#### Scores

The NAFLD Fibrosis Score, APRI Score and Fatty Liver Index [31–33] are useful tools for the diagnosis and monitoring of patients with NAFLD. The NAFLD Fibrosis Score is based on the following parameters: age, Body Mass Index (BMI), whether type-2 diabetes mellitus or impaired fasting glucose, alanine aminotransferase, aspartate aminotransferase, and platelet and albumin levels, while the APRI Score includes the aspartate aminotransferase level and platelet count. The Fatty Liver Index diagnoses fatty liver using laboratory parameters (alanine aminotransferase, aspartate aminotransferase, gamma glutamyl transferase, glucose, insulin, triglycerides, cholesterol levels), and clinical (gender, age, alcohol intake) and anthropometric (waist circumference, triceps, biceps, subscapular and supra-iliac skinfolds) findings. The scores will be calculated via an online calculator (MDCalc.com).

#### Microbiota assessment

For the microbiota assessment, the patients will be instructed to collect a stool sample within 24 h prior to their next visit (baseline and close-out) using a disposable pad and sterile paddle pot. Samples will be immediately frozen in the patient’s own freezer. On the day of the consultation, the patient will transport the sample to the research center using cooling elements and a styrofoam box. Afterwards, samples will be stored at − 80 °C until analysis.

For intestinal microbiota assessment, the bacterial deoxyribonucleic acid (DNA) will be extracted from the feces using the QIAmp DNA Stool Mini kit (Qiagen, São Paulo, Brazil). Extracted DNA will be kept at − 20 °C until the moment of use. Approximately 50 ng DNA will be used for amplification of the V4 hypervariable region of the bacterial *16S* rRNA gene by polymerase chain reaction (PCR). The resulting product will be purified and used in the preparation of the emulsion PCR, followed by the sequencing reaction in the Ion Torrent Personal Genome Machine (Life Technologies, São Paulo, Brazil). The sequencing data will be processed using QIIAME software. Bacterial-diversity analyses will be based on the degree of similarity between *16S* rRNA sequences which are grouped into Operational Taxonomic Units (OTUs). Sequencing and analysis will be performed at the HCPA Protein Analysis Unit (UAMP).

#### Intestinal-permeability assessment

Intestinal permeability will be evaluated by the serum quantification of claudin-3 and LPS biomarkers. From blood samples, serum will be obtained by centrifugation and stored at − 80 °C. The quantification of the biological markers will be performed by enzyme-linked immunosorbent assay (ELISA) according to the manufacturer’s instructions.

#### Sarcopenia

Sarcopenia will be determined following the European Consensus [25] considering muscle mass, muscle strength, and physical performance.

Muscle mass will be measured by the body composition assessment. DEXA (The GE Medical Systems Lunar Prodigy densitometer, Chicago, IL, USA) will be used for body-composition assessment. Fat and lean masses will be determined for the whole body and for body regions (trunk and limbs), as previously described [34]. Electrical bioimpedance (Biodynamics450®, Plainview, NY, USA) will be used to measure the percentage of fat, lean mass, and phase angle. Patients should be fasting for 4 h, not drink alcohol and/or caffeine, not have exercised in the last 12 h, not take diuretics the day before the test, not have a pacemaker in situ, and not be pregnant or menstruating. Patients will be questioned about the presence of significant diarrhea at the time of the examination to exclude the possibility of dehydration [35].

Muscle strength will be evaluated by the handgrip strength (dynamometry). Patients should be seated with their elbow flexed at 90° to hold the dynamometer (Jamar, Duluth, MN, USA) at its maximum strength for 3 s. There will be three repetitions with an interval of 1 min with the dominant hand. The highest strength will be recorded in kgF for classification [36].

Physical performance will be assessed through three different tests: the Five-times sit-to-stand test (FTSTS), the Usual Gait Speed test, and the Unipedal Stance test. The FTSTS test will be performed using a chair leaning against the wall. Initially, participants will be instructed to sit without assistance with their arms crossed in front of the body. If the patient demonstrates being able to perform this task they will be instructed to repeat the test five times, as close together as possible. The test performance will be measured by the time spent running the test [37]. To perform the Usual Gait Speed test the participant must walk a distance of 10 m in a straight line. The time taken to complete the course will be divided by the distance, providing the measure of the speed of the march (m/s). The test will be performed three times and the first and last 2 m will be excluded to discount the acceleration and deceleration phases. Participants will be asked to walk at their normal pace, even using walking aids and no incentive or instruction will be given in order to not influence the results [38]. A speed lower than 0.8 m/s will be considered as risk for sarcopenia [39, 40]. The Unipedal Stance test will be performed to evaluate the patient’s balance. Participants will be instructed to place their hands on their waist and raise one of their legs (chosen by the participant themselves) by flexing the knee and balancing on only one foot for a maximum of 30 s or until the individual becomes unbalanced. The test will be repeated three times and the longest time will be considered [41].

A blood sample will also be collected for the evaluation of testosterone, insulin-like growth factor 1 (IGF-1), and myostatin.

#### Physical-activity assessment

The International Physical Activity Questionnaire – Short Form (IPAQ) will be applied to evaluate the weekly time spent in physical exercise.

#### Cardiovascular-risk assessment

Cardiovascular risk will be estimated by the Framingham Risk Score [42] and the Atherosclerotic Cardiovascular Disease (ASCVD) risk calculator [43].

All data will be included in the database filled out by the researchers, as well as in a physical agenda to log the attendance frequency of the participants. The patients will be contacted to be reminded about the guidelines and to confirm the visits. Subjects who discontinue treatment will also have their data treated at the end of this study.

#### Laboratory assessment

Laboratory assessment will consist of a liver function test (aspartate aminotransferase, alanine aminotransferase, gamma glutamyl transferase, bilirubin, alkaline phosphatase), a lipid profile (triglycerides, total cholesterol, HDL-cholesterol, non-HDL cholesterol, and LDL-cholesterol) and the levels of other relevant indices such as CRP, insulin, glucose, glycated hemoglobin, HOMA-IR, albumin, creatinine, and a blood count. Serum will be obtained by centrifugation and stored at − 80 °C. Quantification of the biomarkers will be performed by ELISA according to the manufacturer’s instructions.

#### Nutritional assessment

The anthropometric assessment will include the measurements of weight and height for calculating BMI, waist circumference between the twelfth rib and the iliac crest with an inextensible fiberglass tape measure, and tricipital, bicipital, subscapular and supra-iliac skin folds using a Lange® adipometer [33].

Dietary intake will be checked by 3-day diet record, describing the foods eaten for three non-consecutive days (two weekdays and one weekend day). The records will be calculated using the NutriBase® software, 2007. Evaluation of total, insoluble, and soluble fiber intake will be calculated through the data of the spreadsheet developed by Schakel et al. [44].

### Data management

The data of the participants will be included in an Excel database and then exported to SPSS version 18. The database will be shared between the data-collecting researchers and a responsible researcher. Patients will be coded according to the order in which they enter the survey following number allocation, corresponding randomization, collection of blood or feces, and begin or end (pre or post treatment). The procedures will be updated in the cadre ClinicalTrials.gov, ID: NCT03467282.

### Statistical analysis

For the quantitative variables, the comparison between the values obtained before and after the intervention will be performed by the *t* test for parametric samples (or Wilcoxon’s signed-rank test if the data do not satisfy the assumptions for this test); for the qualitative variables, the McNemar test will be used.

In the comparison of treatments for the quantitative variables, a delta value will be computed (value before − value after); the delta values will be compared between interventions using the *t* test for independent samples (or the Mann-Whitney *U* test). Delta values can be adjusted by age and other covariates by analysis of covariance. It will be considered statistically significant if *P* < 0.05. Statistical analysis will be performed in SPSS 18.0.

### Data monitoring

There is no significant risk in the supplementation of probiotics, and adverse events or symptoms are rarely described in the literature; therefore, a data monitoring committee is not necessary. However any symptoms will be reported by the patients and considered in this research.

### Harms

In the treatment-adherence control worksheet it will be possible to write down any possible symptoms that may appear during the period. Also, patients will be advised that if they feel any symptom or discomfort during the treatment they will be free to stop.

### Auditing

The research will be regularly reviewed by the responsible researchers and professors involved.

### Confidentiality

The data collected during the surveys will always be treated confidentially. The results will be presented together, without the identification of the participants.

### Ancillary care

In the event of any intercurrent events or damage resulting from the participation of the subjects in the survey, they will receive all necessary care, at no personal cost, at that hospital.

### Dissemination policy

During and after the survey, the ClinicalTrials.gov database will be replete with data. After completing the research and processing the data, an article will be written for later publication in an international journal for the dissemination of data and results.

## Discussion

This is the first double-blind, randomized clinical trial in NASH patients that will evaluate not only the repercussion of 24-week probiotic supplementation on the disease, the intestinal microbiota, and nutritional changes, but also sarcopenic parameters. This study will evaluate several parameters: nutritional, functional, physical, and laboratorial assessments, including tests used for diagnosis and follow-up (TLR-4 and CK-18), and evaluation of degree of liver fibrosis, intestinal permeability, microbiota composition, and cardiovascular risk.

Because NASH is a worrying disease, already considered one of the main indications for liver transplantation [3], it is important to investigate an adjuvant treatment for this disease. It is believed that treating dysbiosis by modulating the intestinal microbiota through probiotic supplementation may be beneficial for NASH patients. However, the studies remain heterogeneous from the point of view of intervention time and products and quantities of supplements used [17–19, 29, 45].

In this study, there is a research team, consisting of dietitian nutritionists, hepatologists, and undergraduate students, committed to the appropriate selection of patients and all data collection. As previously mentioned, the probiotics were donated for this research. We believe that the findings from this study will be generalizable to populations outside Brazil, considering its high methodological quality (based on similar studies), which includes the randomized sample composed of patients found in routine clinical practice.

## Trial status

The study is in the data collection phase. Recruitment started in March 2018 and is predicted to end in May 2019. Forty-five patients have started the study protocol and additional patients are being recruited. The current protocol is version 6.0, created in September 2017 and approved by the Ethics Committee of Hospital de Clínicas de Porto Alegre in October 2017, before randomization.

## Additional file

Additional file 1: Standard Protocol Items: Recommendations for Interventional Trials (SPIRIT) 2013 Checklist: Recommended items to address in a clinical trial protocol and related documents*. (DOC 121 kb)

## Data Availability

Access to data will be controlled by the chief investigator. Donors of probiotics and placebos will have access only to published data. Trial participants or their proxies are provided with assurances about the maintenance of privacy and confidentiality in the informed consent. Participating patients will have access to the results of the routine examinations that are in their medical records, and the other tests will be available from the investigators according to the patients’ interest and request.

## Abbreviations

FTSTS: Five-times sit-to-stand test
kPa: Kilopascals
NAFLD: Non-alcoholic fatty-liver disease
NASH: Non-alcoholic steatohepatitis
PCR: Polymerase chain reaction
TLR: Toll-like receptor
